# Closed isolated anterolateral calcaneal dislocation: a case report

**DOI:** 10.1186/s12891-022-05506-3

**Published:** 2022-06-07

**Authors:** Amir Reza Vosoughi, Armin Akbarzadeh, Alireza Zakaee

**Affiliations:** Department of Orthopedic Surgery, School of Medicine, Shiraz University of Medical Sciences, Shiraz, Iran

**Keywords:** Calcaneus, Calcaneal dislocation, Dislocation, Calcaneocuboid, Subtalar joint

## Abstract

**Background:**

Complete isolated calcaneal dislocation, defined as dislocation of talocalcaneal and calcaneocuboid joints with intact talonavicular joint without significant fracture, is an exceedingly rare injury.

**Case presentation:**

A 49-year-old man, after a motor vehicle collision, presented with a closed isolated anterolateral dislocation of the calcaneus associated with fracture of the sustentaculum tali, cuboid, lateral process of the talus, and avulsion fracture of superior peroneal retinaculum. Urgent successful closed reduction was immediately performed in the emergency room under sedation. Two days later, through sinus tarsi approach extended proximally to posterior of the lateral malleolus and distally to the calcaneocuboid joint, peroneal tendons were reduced in the retromalleolar groove and avulsion fracture of the superior peroneal retinaculum was reduced and fixed by a suture anchor. A chondral lesion (6 × 8 mm) was seen in the posterior facet of the calcaneus for that chondroplasty and microfracture were performed. Also, small bony fragments from the cuboid were excised. The reduced calcaneocuboid joint was fixed by two Kirschner wires. In the second stage, 10 days later, through medial approach to the talocalcaneal joint, multiple free bony fragments were excised. Then sustentaculum tali was reduced and fixed using a lag screw. Postoperatively, a non-weight bearing short leg cast was applied for six weeks. The patient had permission to start partial weight bearing 6 weeks after removal of K-wires. Finally, at the 10th postoperative week, he had full weight-bearing without any aid instrument. He was able to return to his regular activities about 6 months after the injury. At 34 months, the American Orthopaedic Foot and Ankle Society ankle-hindfoot scale was 92 from 100. The foot function index percentile was 7% and the visual analogue score for pain was 1.9. The last radiographs revealed normal configuration of talocalcaneal, calcaneocuboid, and talonavicular joints with a little joint space narrowing in posterior part of the subtalar joint.

**Conclusion:**

Patients with isolated anterolateral calcaneal dislocations, even with multiple associated fractures, can have acceptable outcomes, if it is urgently diagnosed and properly managed.

## Background

The majority of calcaneal fractures are displaced intra-articular subtalar fractures with possible calcaneocuboid joint involvement by fracture lines [1]. Calcaneal fracture-dislocation is a rare injury with displaced and possibly locked posterolateral fragment of the calcaneal fracture in the lateral gutter of the ankle or below the tip of the fibula [2]. It usually associates with fracture of lateral malleolus, ruptured anterolateral ligaments of the ankle, and dislocated peroneal tendons with rupture or avulsion fracture of superior peroneal retinaculum [3]. Calcaneal fracture-dislocation should be differentiated from isolated calcaneal dislocation, an exceedingly rare injury, which has been rarely reported in the literature [4–6].

Isolated calcaneal dislocation is defined as subluxation or dislocation of the talocalcaneal and calcaneocuboid joints with intact talonavicular joint [5]. Notably, for being dislocated from talocalcaneal joint, calcaneus should be freed from medial constant fragment, sustentaculum tali. Major fracture line, like what we often see in calcaneal fractures, extended to anterior part of calcaneus should not be seen in isolated calcaneal dislocations. Despite rarity of isolated calcaneal dislocation, it may lead to poor outcome, if it is not urgently diagnosed and properly managed [5]. We are going to present a rare case of complete isolated anterolateral dislocation of the calcaneus with description of the outcome after 34 months.

## Case presentation

**Fig. 1:**
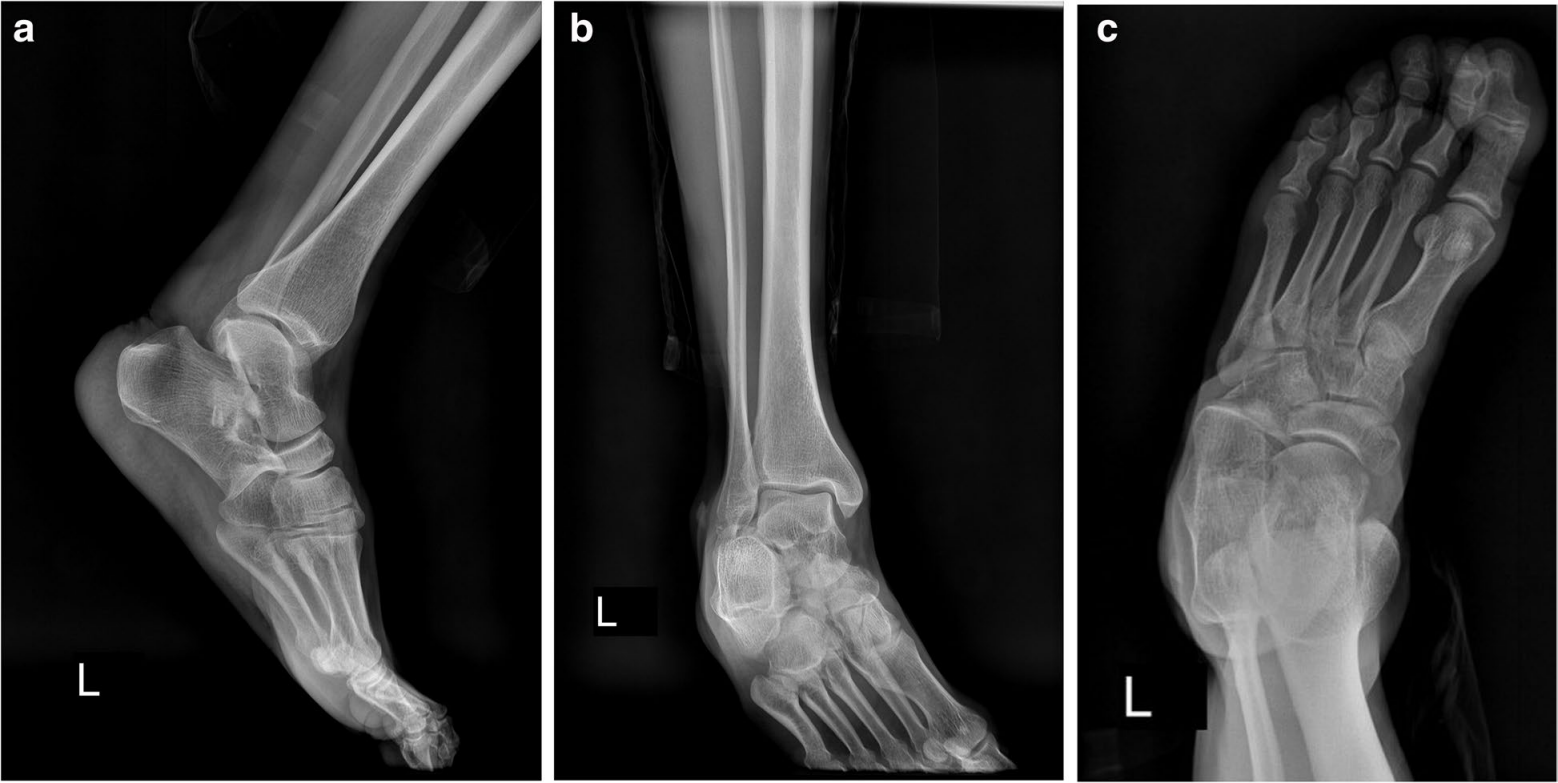
Anterolateral dislocation of the talocalcaneal and calcaneocuboid joints with intact talonavicular joints. Lateral (**a**) and anteroposterior (**b**) views of the ankle joint, oblique radiograph of the foot (**c**)

**Fig. 2:**
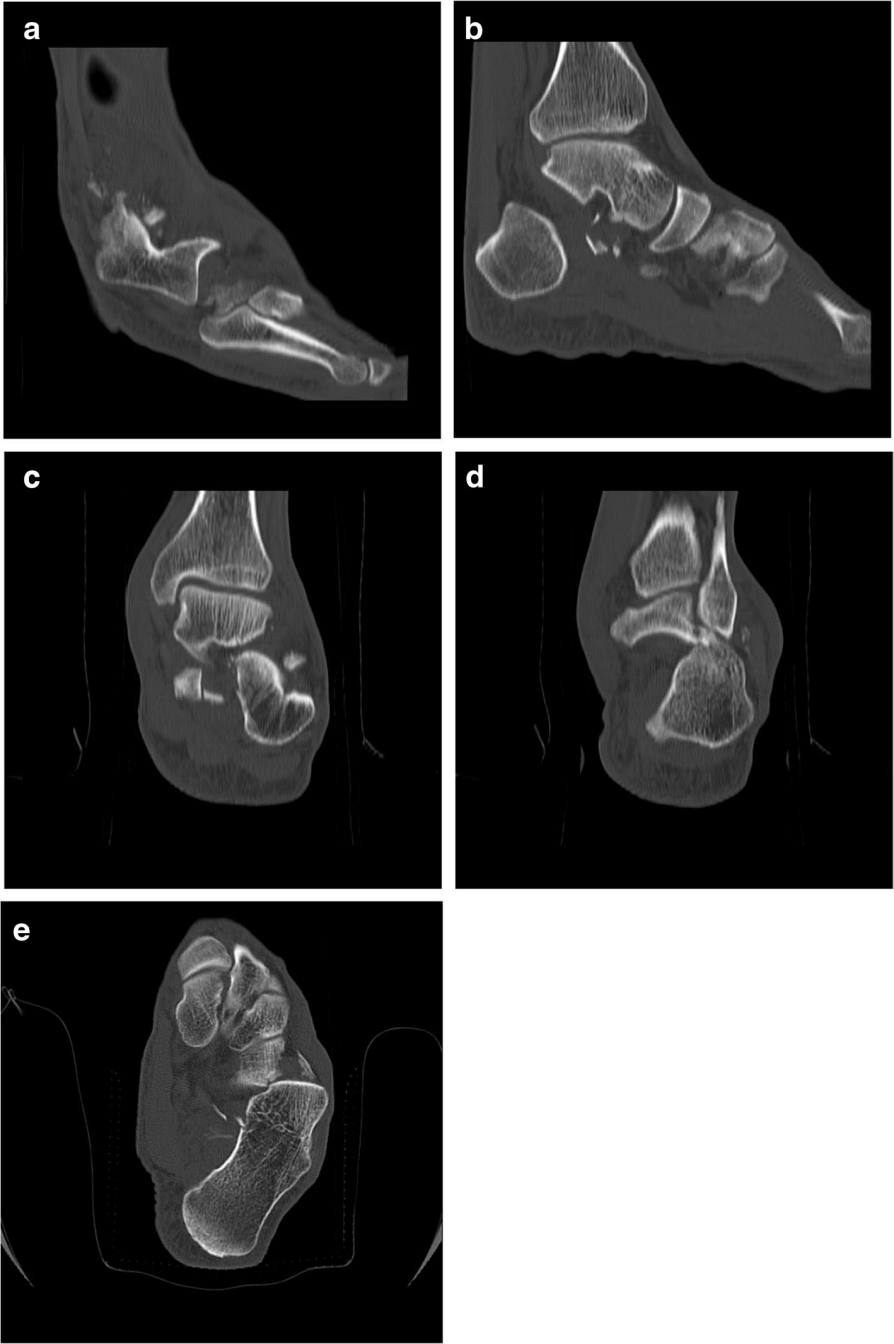
Pre-reduction sagittal (**a** & **b**), coronal (**c** & **d**) and axial (**e**) CT scan showed isolated anterolateral dislocation of the calcaneus

**Fig. 3:**
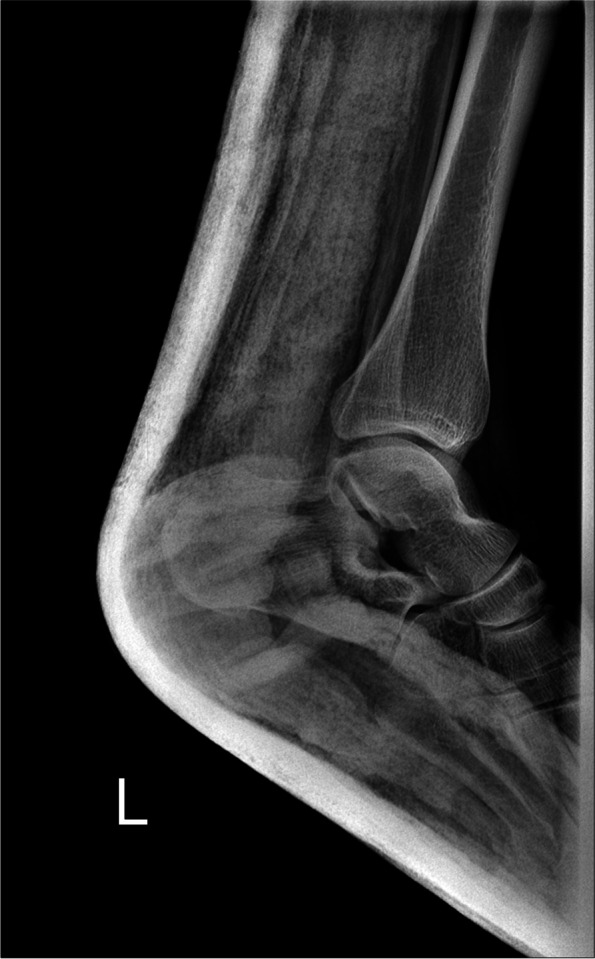
Lateral radiograph of the ankle after closed reduction in the emergency department

**Fig. 4:**
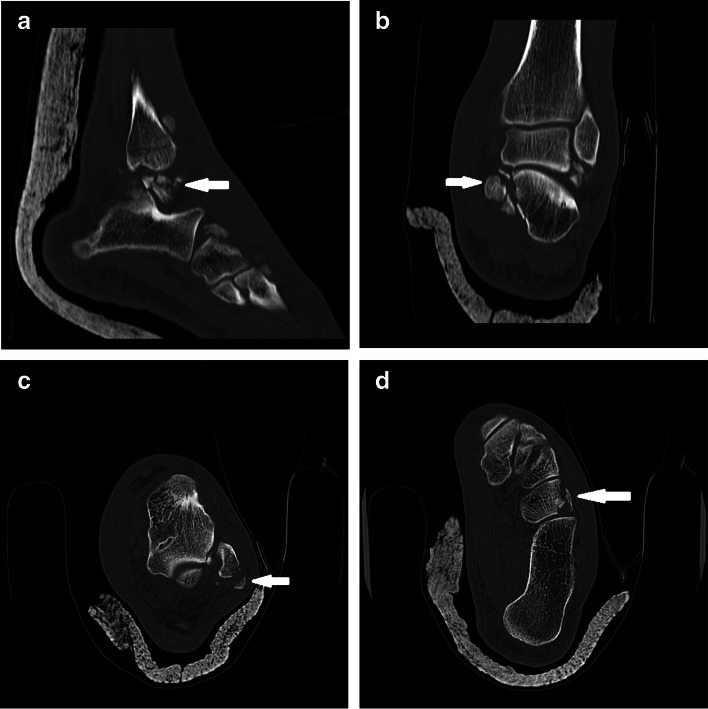
Post-reduction CT scan showed Comminuted fracture of lateral process of the talus (**a**), the sustentaculum tali (**b**), avulsion fracture of superior peroneal retinaculum (**c**) with dislocated peroneal tendons, and cuboid (**d**)

A 49-year-old man referred to the emergency department following a high-energy trauma in a motor vehicle collision. On arrival, the ankle was severely painful with swelling and a little ecchymosis. The patient did not have concomitant injuries. Physical examination revealed intact arterial supply of the foot without neurological deficit or skin laceration. Initial plain radiographs revealed dislocation of talocalcaneal and calcaneocuboid joints with comminuted avulsion fragments on the tip of the lateral malleolus. It appeared that talonavicular joint was intact (Fig. 1). A computed tomography (CT) scan was requested to delineate the injury pattern (Fig. 2). Because of swelling and deformity, the successful simple reduction was immediately performed by traction of the toes and directly pushing the calcaneus medially to its anatomical position, in the emergency room under sedation. The lower limb was placed in a short leg backslab (Fig. 3) and the reduction was confirmed with a post-reduction CT scan. Comminuted displaced fracture of the sustentaculum tali, cuboid, lateral process of the talus, and avulsion fracture of superior peroneal retinaculum were apparent (Fig. 4).

**Fig. 5:**
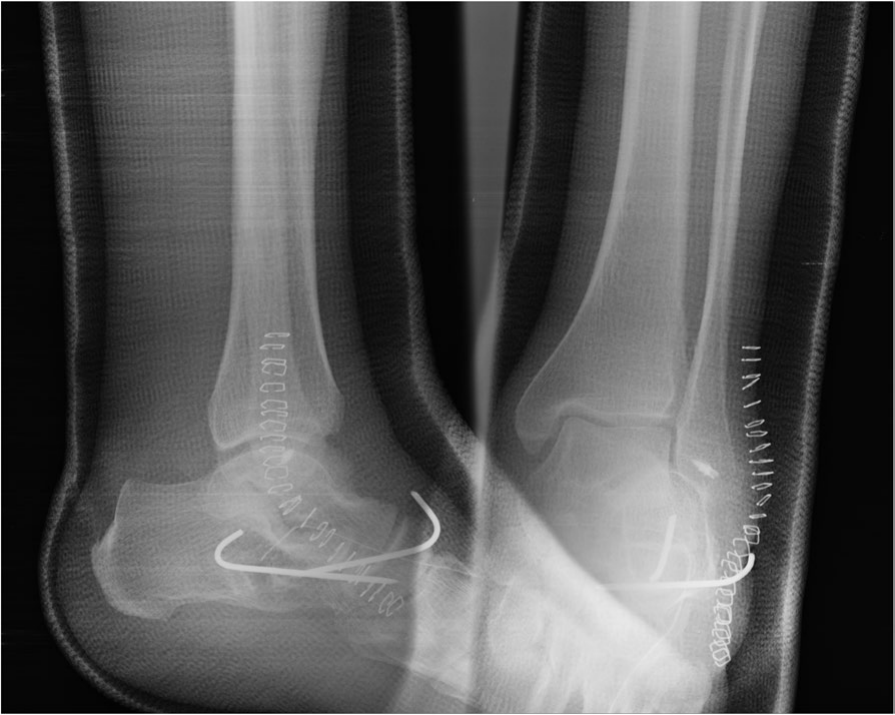
Radiographs of the ankle after removal of free fragments, fixation of superior peroneal retinaculum avulsion, and reduction and fixation of the calcaneocuboid joint

Two days after the injury, through sinus tarsi approach extended proximally to posterior of the lateral malleolus and distally to the calcaneocuboid joint, arthrotomies were done and multiple free osteocartilaginous fragments from lateral process of the talus were excised. A chondral lesion (6 × 8 mm) was seen in the posterior facet of the calcaneus for that chondroplasty and microfracture were performed. The avulsion fracture of the superior peroneal retinaculum was reduced and fixed to its anatomical position by a suture anchor, after reduction of the peroneal tendons in the retromalleolar groove. Also, small bony fragments from the cuboid were excised. Based on Fig. 4d, about 60% of proximal articular surface of cuboid was intact and after perfect reduction of the calcaneocuboid joint, there was no any risk of lateral column instability or shortening; therefore, calcaneocuboid joint was fixed by two Kirschner wires in order to stabilize it (Fig. 5). Because talocalcaneal joint was stable by stress tests under guide of fluoroscopy, no Kirschner wire was inserted in it. Due to bullae formation in medial of the ankle, we decided to postpone reduction and fixation of the sustentaculum tali.

In the second stage, 10 days later, through the medial approach to the talocalcaneal joint, by retracting flexor hallucis longus tendon downward, the fractured sustentaculum tali was exposed. It was partially detached from surrounding soft tissues and displaced in order to see intraarticular and extraarticular free fragments. After subtalar joint arthrotomy, multiple free bony fragments were excised. Then sustentaculum tali was reduced and fixed using a lag screw. The stability of subtalar joint was checked by stress view under guide of fluoroscopy.

Postoperatively, a non-weight bearing short leg cast was applied for six weeks. Then K-wires were removed and physiotherapy was started. The patient had permission to start partial weight bearing 6 weeks following the second surgery. Finally, at the 10th postoperative week, he had full weight-bearing without any aid instrument.

**Fig. 6:**
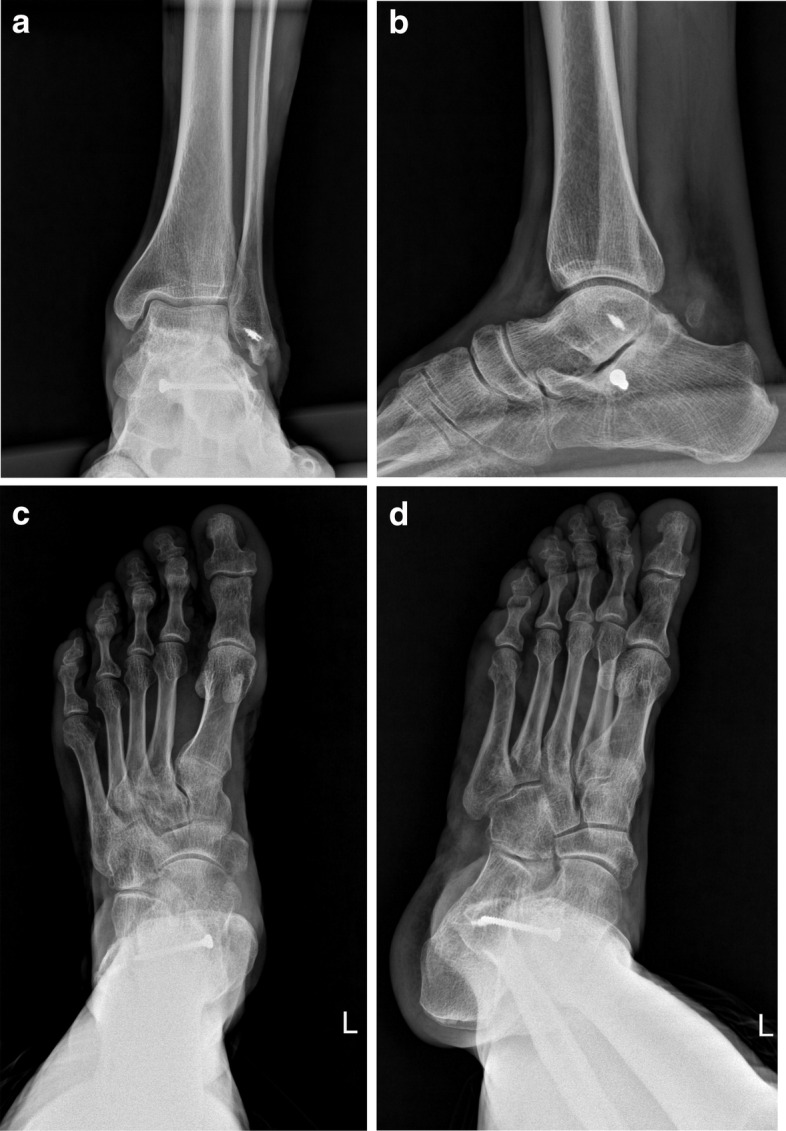
Radiographs of the patient at 34 months after the injury

At 34 months after the injury, the patient was recalled for a follow-up visit. He was able to return to his regular activities about 6 months after the injury. The function was evaluated using the American Orthopaedic Foot and Ankle Society (AOFAS) ankle-hindfoot scale [7], the foot function index (FFI), and visual analogue score for pain (VAS). The total AOFAS ankle-hindfoot scale was 92 from 100 (no pain, moderate restriction in hindfoot motion and mild hindfoot valgus deformity). The FFI percentile was 7% and the VAS score of the pain was 1.9. The last radiographs revealed normal configuration of talocalcaneal, calcaneocuboid, and talonavicular joints with a little joint space narrowing in posterior part of the subtalar joint (Fig. 6).

Informed written consent was obtained from the patient for publication.

## Discussion and conclusion

There are different kinds of dislocation in the ankle, hindfoot, and Chopart joints. Isolated calcaneal dislocation should be differentiated from subtalar or pantalar dislocations, in which talonavicular subluxation or dislocation is a featured injury [8]. Isolated dislocation of the calcaneus from talocalcaneal and calcaneocuboid joints is a very rare injury [4–6]. Also, this entity should be distinguished from calcaneal fracture-dislocation, a subtype of displaced intra-articular calcaneal fracture, in which the posterolateral fragment of posterior facet of the calcaneus placed and locked in the lateral gutter of the ankle or below tip of the fibula. In calcaneal fracture-dislocations, calcaneocuboid dislocations or fracture-dislocation should not be seen; however, the line of fracture may be extended to the calcaneocuboid joint. These patients usually present with marked hindfoot swelling, varus tilt of the talus, and fracture of the lateral malleolus [2, 3]. These cases usually have dislocation of the peroneal tendons and most of them have Sanders type IIB calcaneal fractures [9]. Calcaneal fracture-dislocation should be surgically approached similar to other displaced intra-articular calcaneal fractures through lateral approaches using plate and/or lag screws [9].

On the other hand, an isolated calcaneal dislocation is not associated with locked posterolateral fragment of the calcaneus in the lateral part of the ankle joint. Posterior facet is completely detached from sustentaculum tali without any fracture line in the other parts of the calcaneus, except fracture-dislocations of the calcaneocuboid joint. Fracture of the anterior process of calcaneus is the result of impaction in the calcaneocuboid joint at the time of calcaneocuboid subluxation or dislocation. Anterior process fracture may not be seen in all cases of isolated calcaneal dislocation, such as our case. The surgical approach in isolated calcaneal dislocations is different from calcaneal fracture-dislocation. It is not necessary to fix the calcaneal fracture by a plate and/or lag screws from lateral side via sinus tarsi approach or lateral extensile approaches. Reduction and fixation of sustentaculum tali from medial side is a key point.

By extensive review of the English-language literature, we summarized previous reported cases in Table 1. Some previous reports were not isolated calcaneal dislocation because of subluxation of talonavicular joints [10], hence we did not add them in Table 1. Moreover, there were several case reports mentioned in the previous studies [6] without definite description of the case or apparent radiographs. These are as follows,

1. Inferior dislocation of the calcaneus in a patient refused treatment [11].
2. Lateral subluxation of the calcaneus without tarsal bone fractures in a 54-year-old man [12].
3. Lateral dislocation of the calcaneus in a 16-year-old girl [13].

**Table 1:** Previous reported cases of isolated calcaneal dislocation

Author(s)	Sex	Age	Mechanism	Type	Associated injuries	Treatment	Follow up time (months)	outcome
Northover & Milner (4)	M	33	Motor vehicle collision	Closed, Anterolateral	Fracture of anterior process & sustentaculum tali	Closed reduction & pinning	6	Symptom free
Nepple et al. (5)	M	45	Motorcycle accident	Closed, Anterolateral	Fracture of anterior process & sustentaculum tali	Triple arthrodesis (one month delay in treatment)	12	Osteomyelitis & wound dehiscence finally: heel varus with mild discomfort
Nepple et al. (5)	M	35	Motorcycle accident	Closed, Anterolateral	Fracture of sustentaculum tali, cuboid & posterior talar process	2 stages: 1) External fixator 2) ORIF Cuboid & Nonoperative treatment of sustentaculum tali fracture	6	Mild pain on uneven surfaces
Nepple et al. (5)	F	28	Motor vehicle collision	Open, Anterolateral	Fracture of anterior process, sustentaculum tali, medial cuneiform, navicular, second metatarsal, third metatarsal & fibula	Irrigation and debridement, Dual spanning external fixators	12	Stiffness & minimal intermittent right foot discomfort
Vishwanath & Shephard (6)	F	22	Motor vehicle collision	Closed, Anterolateral	Not mentioned	Open reduction & calcaneocuboid joint fixation with K-wire	30	Limping, mild varus, equinus & supination deformity
Our case	M	49	Motor vehicle collision	Closed, Anterolateral	Fracture of sustentaculum tali, cuboid, lateral process of talus & superior peroneal retinaculum avulsion fracture of fibula	3stages; 1) Closed reduction 2) Open reduction & pinning of calcaneocuboid joint 3) Open reduction and fixation of sustentaculum tali	34	AOFAS: 92 VAS pain: 1.9 Return to pre-injury level of activity

By studying Table 1, we can conclude that isolated calcaneal dislocations are the result of high-energy trauma, usually after motor vehicle accidents. Fracture of sustentaculum tali is seen in all cases, because medial support of the calcaneus, constant fragment, should be detached from the other parts in order to have anterolateral dislocation of the calcaneus. Moreover, over anterolateral dislocation of the calcaneus, the impact between anterior process of the calcaneus and the cuboid may lead to comminuted fracture of these parts. The majority of these cases had closed injuries (5 out of 6 cases) with associated comminuted fracture of the anterior process of the calcaneus [4, 5]. Our case had no fracture of anterior process of the calcaneus but cuboid, similar to one of previous reported cases [5].

Isolated anterolateral dislocation of the calcaneus usually occurs when an axial load is applied on the hindfoot in varus posture. This may lead into a shearing force of the talus on the sustentaculum tali that may lead to the fracture of sustentaculum tali. For lateral dislocation of the calcaneus, the surrounding ligaments are needed to get disrupted [14].

Based on our case and other reported cases in the literature, the following points should be considered in management of isolated calcaneal dislocations.

- Closed reduction should be urgently attempted in the emergency department due to simple reducibility of these cases.
- Open reduction and fixation of calcaneal anterior process fractures, cuboid fractures, and sustentaculum tali fractures are essential parts of the management.
- Stability of talocalcaneal and calcaneocuboid joints should be evaluated after reduction. K-wires or external fixators can be used for keeping them stable.
- Free osteocartilaginous fragments in the joints should be removed to reduce the chance of future arthrosis.
- Shortening of lateral column of foot should be assessed carefully. Sometimes external fixator or spanning temporary plates are necessary.
- In old cases, triple arthrodesis is an option.

In conclusion, patients with isolated anterolateral calcaneal dislocations, even with multiple associated fractures, can have acceptable outcomes, if it is urgently diagnosed and properly managed.

## Data Availability

Not applicable.

## Abbreviations

AOFAS: American Orthopaedic Foot and Ankle Society
FFI: Foot function index
VAS: Visual analogue score
CT: Computed tomography
